# Paroxysmal Kinesigenic Dyskinesia: Genetics and Pathophysiological Mechanisms

**DOI:** 10.1007/s12264-023-01157-z

**Published:** 2023-12-13

**Authors:** Jiao-Jiao Xu, Hong-Fu Li, Zhi-Ying Wu

**Affiliations:** 1 Department of Medical Genetics and Center for Rare Diseases, The Second Affiliated Hospital, Zhejiang University School of Medicine, Hangzhou, 310009 China; 2 Department of Neurology in the Second Affiliated Hospital, Key Laboratory of Medical Neurobiology of Zhejiang Province, Zhejiang University School of Medicine, Hangzhou, 310009 China

**Keywords:** Paroxysmal kinesigenic dyskinesia, *PRRT2*, *TMEM151A*, Genetics, Pathophysiological mechanisms

## Abstract

Paroxysmal kinesigenic dyskinesia (PKD), the most common type of paroxysmal movement disorder, is characterized by sudden and brief attacks of choreoathetosis or dystonia triggered by sudden voluntary movements. PKD is mainly caused by mutations in the *PRRT2* or *TMEM151A* gene. The exact pathophysiological mechanisms of PKD remain unclear, although the function of PRRT2 protein has been well characterized in the last decade. Based on abnormal ion channels and disturbed synaptic transmission in the absence of PRRT2, PKD may be channelopathy or synaptopathy, or both. In addition, the cerebellum is regarded as the key pathogenic area. Spreading depolarization in the cerebellum is tightly associated with dyskinetic episodes. Whereas, in PKD, other than the cerebellum, the role of the cerebrum including the cortex and thalamus needs to be further investigated.

## Introduction

Paroxysmal kinesigenic dyskinesia (PKD) is a paroxysmal movement disorder characterized by brief and recurrent episodes of dystonic or choreoathetotic movements triggered by sudden voluntary movements or startle [1]. Attacks last less than one minute, and consciousness is usually normal for the whole episode. Antiepileptic drugs can effectively control attacks. PKD frequently begins in childhood or early adolescence and many patients report decreased attacks during adulthood. Male patients are far more numerous than female patients, with a ratio of 2–4:1 [2]. In the general population, PKD is rare, with the prevalence estimated at 1:150,000 [3]. Based on the etiology, PKD can be divided into primary PKD and secondary to underlying causes such as lesions of the central nervous system or metabolic disorders. The major cause of primary PKD, both sporadic and familial, is genetic mutations [2]. Familial PKD mainly exhibits an autosomal dominant pattern of inheritance. In the past decades, two genes, *PRRT2* and *TMEM151A*, have been described as the causative genes for PKD [4, 5]. Occasionally, mutations in other genes such as *KCNA1* and *SLC2A1* have been detected in rare PKD patients [6, 7]. Among them, the *PRRT2* gene was the first identified causative gene and accounts for the majority of PKD cases [4]. Li *et al.* reported that PKD with *PRRT2* mutation are likely to achieve complete remission through treatment with a low dose of carbamazepine (CBZ). Although CBZ is adequate to provide good control of the symptoms, most PKD patients without *PRRT2* mutations do not achieve remission after obtaining an increased dose of CBZ [8]. Besides, adverse effects of medication are present in a subset of PKD patients. Studies in the pathophysiological mechanisms of PKD are required to prompt the development of more targeted therapeutic methods. Disturbance of ion channels and synaptic transmission have been repeatedly found in research and PKD is suspected to be a channelopathy or a synaptopathy [9–11]. However, the pathogenesis of PKD is complex, and current knowledge cannot explain it well. In this narrative review, we discuss the genetics and pathophysiological mechanisms of PKD, with a focus on recent advances.

## Genetics

### Mutations in* PRRT2*

**Fig. 1:**
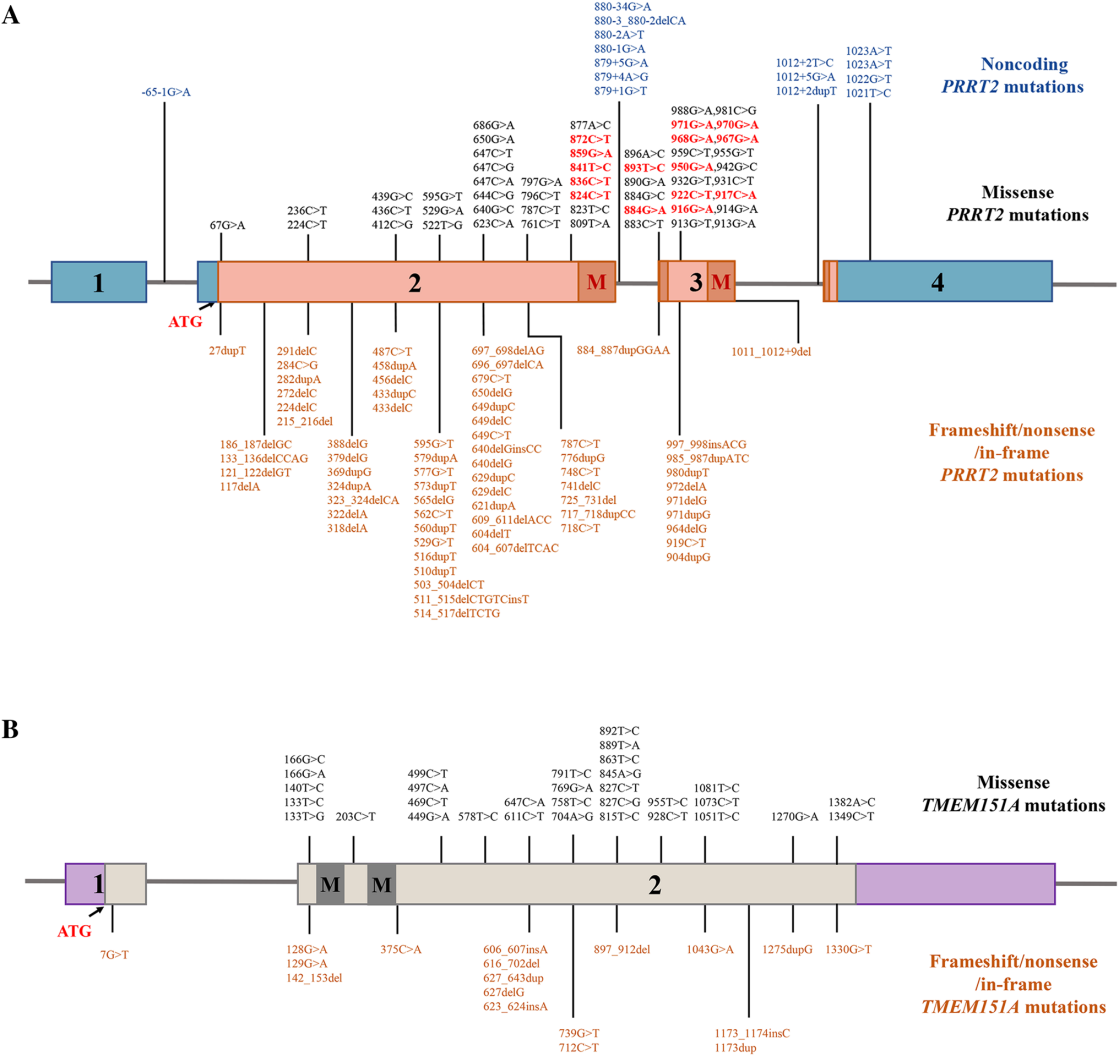
Mutations in the *PRRT2* and the *TMEM151A* gene*.* **A** Schematic drawing of the *PRRT2* gene containing four exons. All *PRRT2* mutations in HGMD including missense mutations (black), frameshift/nonsense/in-frame mutations (orange), and noncoding mutations (blue) are visualized in the *PRRT2* gene. Among missense mutations, likely pathogenic mutations were indicated in red characters. M: transmembrane region. **B** Schematic drawing of the *TMEM151A* gene and diagram of reported mutation.

In 2011, *PRRT2* was identified as the first causative gene of PKD [4], which was subsequently confirmed in different ethnic groups [12]. The *PRRT2* gene is located on human chromosome 16p11.2 and consists of four exons. More than 100 mutations in *PRRT2* (Fig. 1A) have been described according to the Human Gene Mutation Database (HGMD, https://www.hgmd.cf.ac.uk/). The vast majority of *PRRT2* mutations are truncated mutations including nonsense and frameshift mutations, leading to a premature stop codon. Among them, c.649dupC, accounting for up to 80% of *PRRT2* mutation carriers [13], is a high-frequency mutation and possibly arises from *de novo* mutagenesis in sporadic PKD [14]. An unstable DNA sequence, 4 guanine bases followed by a homopolymer tract of 9 cytosine bases where c.649dupC occurs, has been proposed to be the reason for the high frequency of this mutation [9]. Pathogenic or likely pathogenic missense mutations and intronic mutations represent only a small percentage. Moreover, a 16p11.2 microdeletion including the *PRRT2* gene has also been described in a few isolated PKD patients [15]. Copy number variants can be considered when point mutations in *PRRT2* are not found in PKD, especially in sporadic patients.

Functional study and the pathogenicity classification of *PRRT2* missense mutations indicate that all the likely pathogenic mutations cluster at the C-terminus of PRRT2 (Fig. 1A). The *PRRT2* gene encodes a 340-amino-acid transmembrane protein with a proline-rich domain in the large intercellular N-terminal region and two putative transmembrane domains at the C-terminal region. Pathogenic missense mutations lead to subcellular mis-localization of PRRT2 from the plasma membrane to the cytoplasm [16, 17]. This mislocation might be related to damaged transmembrane domains located at the C-terminus. In addition, truncated mutations without a complete C-terminus cause decreased PRRT2 protein levels associated with proteasome-mediated truncated protein degradation [18]. The defective C-terminus of PRRT2 is strongly correlated with PKD.

**Fig. 2:**
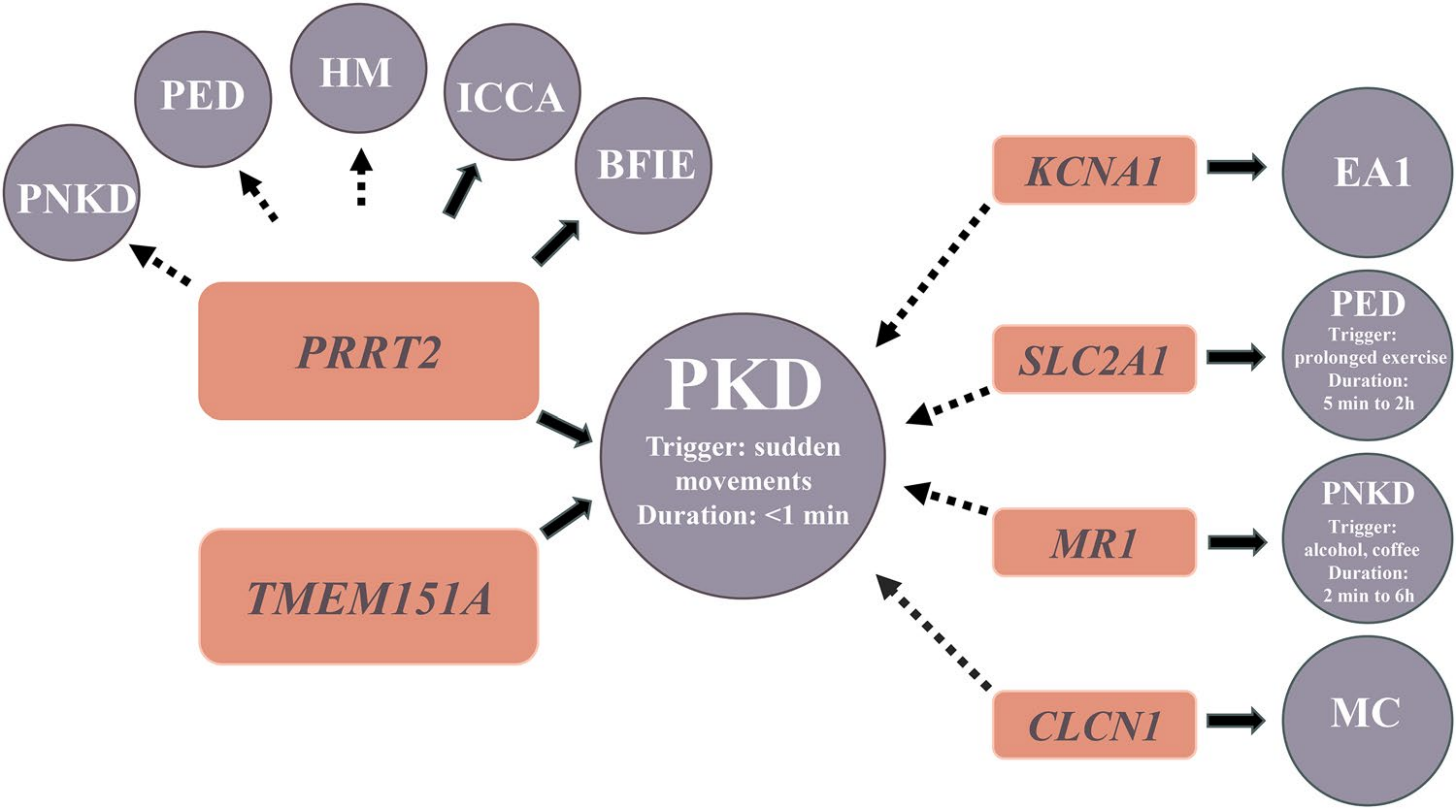
Associated genes and the wide clinical spectrum of associated clinical features with PKD. The circles indicate clinical phenotype**,** the squares indicate genes, the filled arrows indicate a definite association between gene and clinical phenotype, and the dotted arrows indicate an uncertain association between gene and clinical phenotype. PKD: paroxysmal kinesigenic dyskinesia, PNKD: paroxysmal nonkinesigenic dyskinesia, PED: paroxysmal exercise-induced dyskinesia, HM: hemiplegic migraine, ICCA: infantile convulsions with choreoathetosis, BFIE: benign family infantile epilepsy, EA: episodic ataxia, MC: myotonia congenita.

Studies of the genotype-phenotype correlation have revealed that PKD patients with *PRRT2* mutations tend to present with an earlier onset, a longer attack duration, the choreoathetosis phenotype, bilateral limb involvement, and more forms of dyskinesia than patients without *PRRT2* mutations [8, 13, 19]. However, the clinical symptoms of patients with *PRRT2* mutations show high heterogeneity. PKD is the major phenotype of *PRRT2* mutations. Notably, not only PKD but also other neurological disorders including benign family infantile epilepsy [20] and infantile convulsions with choreoathetosis [21], are associated with *PRRT2* mutations (Fig. 2). Occasionally, *PRRT2* mutations have also been implicated in other paroxysmal neurological disorders, such as hemiplegic migraine [22], paroxysmal nonkinesigenic dyskinesia (PNKD), and paroxysmal exercise-induced dyskinesia (PED) [23]. Indeed, family members carrying identical *PRRT2* mutations may exhibit variable phenotypes. In a pedigree with c.649dupC mutation, the proband had transient infantile paroxysmal torticollis followed by BIE, his father developed PKD and migraine, and his brothers presented hemiplegic migraine [24]. Of note, quite a few people carrying pathogenic *PRRT2* mutations have no symptoms. According to the latest statistics, the penetrance of *PRRT2* mutations is about 74.5% to 77.6% [25]. Modifier genes, epigenetic modification, and environmental factors may be associated with the remarkable phenotypic variability and incomplete penetrance.

Heterozygous *PRRT2* mutations leading to loss-of-function of PRRT2 and haploinsufficiency in PKD are widely believed. Homozygous or compound heterozygous *PRRT2* mutations which have been reported rarely may cause a complete lack of normal function. Indeed, clinical studies demonstrated that patients with biallelic *PRRT2* mutations exhibited more severe phenotypes including multiple paroxysmal disorders, prolonged ataxia attacks, and intellectual disability [26, 27]. Mild development delay is also frequent in those PKD patients with 16p11.2 microdeletion [15], while PKD patients with heterozygous *PRRT2* mutations were rarely reported as obviously lacking in intelligence. Thus, more research is needed to determine the relationship between the phenotype of development delay and PRRT2.

### Mutations in *TMEM151A* and other Genes

To date, *PRRT2* mutations account for 69% to 93% of familial PKD and 21% to 45% of sporadic PKD [13, 28, 29]. A certain percentage of PKD patients without *PRRT2* mutations prompts researchers to find other causative genes. In 2021, Li *et al.* identified that mutations in *TEME151A* cause PKD in Chinese cohorts without *PRRT2* mutations [5], which was verified in different populations [30, 31]. *TMEM151A* is a poorly characterized gene located on chromosome 11q13.2 and consists of two exons. The full-length TMEM151A protein contains 468 amino acid residues with two putative transmembrane domains (amino acids 45–65 and 98–118). To date, more than 50 *TMEM151A* mutations (Fig. 1B), including truncated mutations, missense mutations, and non-frame deletion, have been identified in PKD cases. In recent studies, non-truncated mutations cause decreased TMEM151A protein *in vitro* study, and transcript analysis of PKD patients with the c.606_607insA mutation revealed a half reduction in TMEM151A mRNA [32]. These studies indicate that loss of function and haploinsufficiency may be the pathogenic mechanism underlying *TEME151A*-linked PKD. Compared with PKD patients with *PRRT2* mutations, patients with *TMEM151A* mutations are more common in sporadic cases, which could be attributed to lower penetrance or *de novo* mutagenesis [29]. The penetrance of *TMEM151A* mutations was estimated to be about 53.8%, which was lower than that of *PRRT2*. Besides, *TMEM151A-*linked PKD patients tend to present a pure phenotype, a shorter duration of attacks, and a dystonia phenotype [29, 33]. They also respond well to CMZ or oxcarbazepine (OXC) with decreased attacks. Whereas, the response tends to be incomplete in *TMEM151A-*linked PKD, with occasional attacks or sensory aura remaining. We suppose that the variable degree of symptoms between these two genes associated-PKD may be caused by different mechanisms.

In addition, pathogenic mutations in *KCNA1*, the major causative gene for episodic ataxia 1, were also reported in familial PKD without *PRRT2* mutations. Electrophysiological examinations showed that two heterozygous *KCNA1* mutations in PKD patients caused the K_v_1.1 channel dysfunction [7]. Besides, other mutant genes were also reported in a few PKD patients without *PRRT2* mutations, such as *MR-1*, *SLC2A1*, and *CLCN1* (Fig. 2), which are causative genes for PNKD, PED, and myotonia congenita (MC), respectively [6]. However, up to date, no powerful evidence has suggested that these genes are causative genes for PKD. One possible reason is that the mutant genes of these PKD patients were analyzed without consideration of the 16p11.2 microdeletion and *TMEM151A.* Although carrying pathogenic mutations in these genes, incomplete penetrance should certainly be considered in patients. Then, phenotypic overlap may lead to difficulty in distinguishing PKD from other paroxysmal movement disorders and result in misdiagnosis. Paroxysmal movement disorders have similar attacks but different trigger factors and duration of attacks. Since MC and PKD share some clinical features, cautious consideration in diagnosis is also needed. They both present with stiffness or muscle weakness which are pronounced on an initial action. It is worth mentioning that a Chinese patient had a predominant phenotype of choreoathetosis attacks and additional signs of myotonia, with the coexistence of a *PRRT2* mutation and two *CLCN1* mutations [34]. Notably, myotonic discharges in the EMG examination can be detected in MC patients, but not in PKD. Thus, when patients are clinically diagnosed with PKD, an additional EMG test and screening of the *CLCN1* gene would be needed to exclude MC. Thus, no sufficient evidence improves these mutant genes associated with the occurrence of PKD.

## Pathophysiologic Mechanisms

### Disturbed Signal Transmission in Neurons

The paroxysmal manifestations observed in PKD patients suggest network instability in the neural system. The major causative *PRRT2* gene encodes a transmembrane protein in neurons but not neuroglia cells. The neuron is a basic structural and functional unit in the neural system, transmitting signals through two modes including spiking (action potential) and synaptic transmission. Recent studies have shown that PRRT2 interacts with several proteins (Table 1) which are involved in the process of signal transmission. Loss-of-function of PRRT2 protein in transmitting signal may cause network instability and lead to PKD.

**Table 1:** List of the proteins reported to interact with PRRT2

Interacting proteins	Type	References
SNAP25	Synaptic protein	Lee *et al.* [21]
SNAP25, GRIA1	Synaptic protein	Li *et al.* [41]
SNAP25	Synaptic protein	Liu *et al.* [50]
Intersectin1	Synaptic protein	Rossi *et al.* [49]
SNAP25, VAMP2, Syt1, Syt2	Synaptic protein	Valente *et al.* [42]
STX1A, SNAP25	Synaptic protein	Tan *et al.* [18]
VAMP2, SNAP25, STX1A, Syt1	Synaptic protein	Coleman *et al.* [46]
Na_v_1.2/Na_v_1.6 channel	Ion channel	Fruscione *et al.* [11]
SNAP25, STX1A, VAMP2, GRIA1	Synaptic protein	Mo *et al.* [48]
P/Q-type Ca^2+^ channel	Ion channel	Ferrante *et al.* [47]
Na^+^/K^+^ ATPase pump (NKA)	Ion transporter	Sterlini *et al.* [35]

GRIA1: glutamate ionotropic receptor AMPA type submit 1, VAMP2: synaptobrevin-2, STX1A: syntaxin1a, Syt1: synaptotagmin-1, Syt2: synaptotagmin-2.

**Fig. 3:**
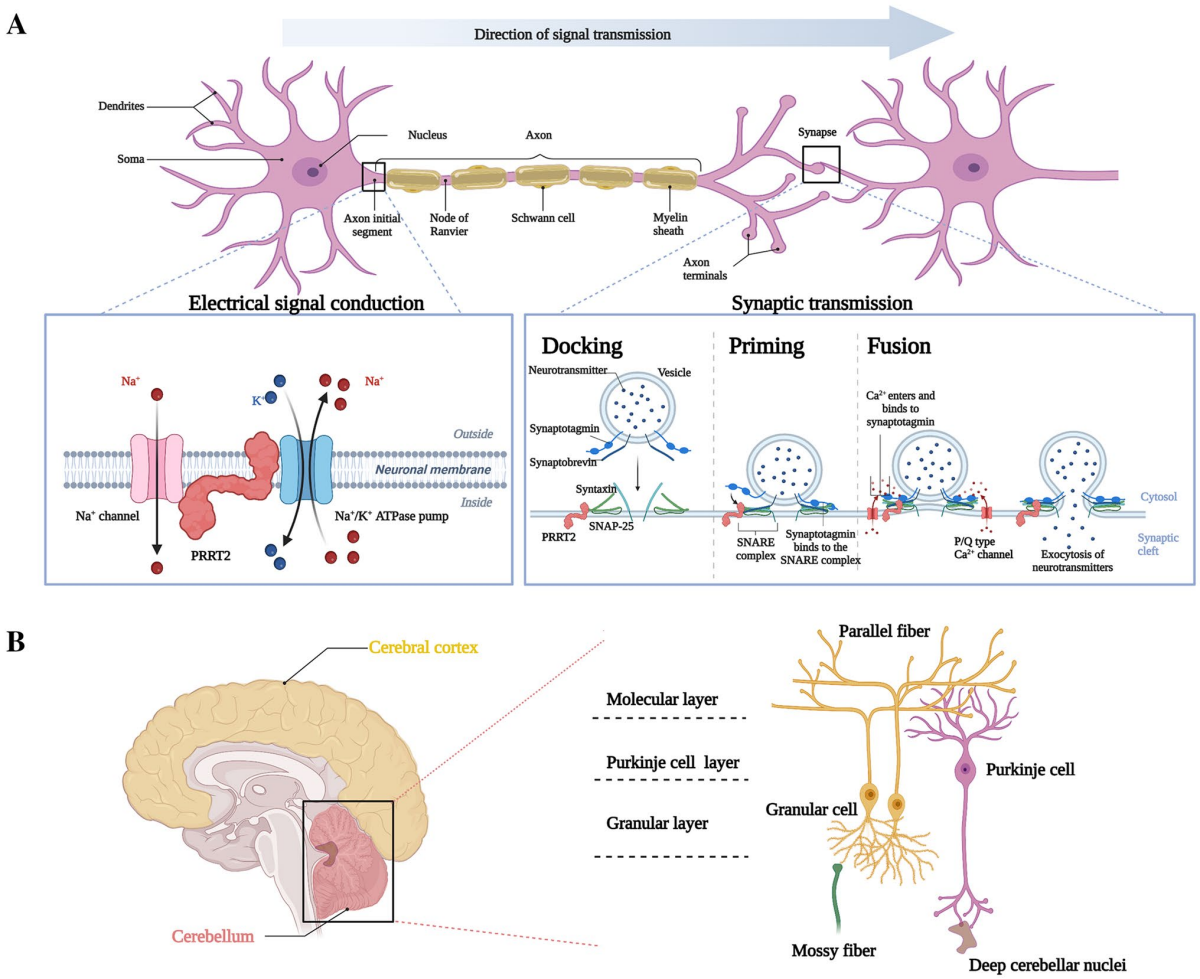
Pathophysiological mechanisms of PKD. **A** PRRT2 influences signal transmission in neural systems including electric signal conduction in neurons and synaptic transmission between neurons. In electric signal conduction, two types of protein, the ion transporter Na^+^/K^+^ ATPase pumps, and the voltage-gated Na_v_1.2/Na_v_1.6 channels, are involved in the electric signal conduction and interact with PRRT2 in neurons. Na^+^/K^+^ ATPase pumps located on the neuron maintain the ion gradient and the voltage-gated Na_v_1.2/Na_v_1.6 channels located at the axon initial segment (AIS) are responsible for the generation of action potentials. In synaptic transmission, PRRT2 is involved in the three-step process of neurotransmitter release by interacting with various presynaptic proteins: (1) synaptic vesicle (SV) docking at the active zone (AZ); (2) SV priming and forming the SNARE complex (syntaxin, SNAP25 and synaptobrevin) assembly; (3) the Ca^2+^ sensor, synaptotagmin, triggers synchronous SV fusion due to the concomitant increase in the intracellular Ca^2+^ concentration. **B** The pathogenic key area is in the cerebellum and the granule cell (GC)-Purkinje cell (PC)-deep cerebellar nuclei (DCN) pathway is the deficit in the cerebellum. The cerebellar cortex is made up of three layers: the molecular layer (outer layer), the Purkinje cell layer (median layer), and the granular layer (inner layer). The parallel fibers of GCs and dendrites of PCs form synaptic connections in the molecular layer. PCs send inhibitory projections to the DCN.

PKD was proposed to be an ionic channelopathy due to the episodic nature of the symptoms. Meanwhile, most PKD patients exhibit a favorable response to antiepileptic drugs, such as CMZ and OXC, all of which modulate different types of ion channels. The paroxysmal clinical manifestation and excellent response to antiepileptic drugs of PKD may be associated with abnormal ion channel functions *in vivo*. According to research findings, PRRT2 influences neural excitability by interacting with ion channels in neurons (Fig. 3A) [11, 35]. Various ion channels, such as Na^+^ channels and K^+^ channels, are at the membrane surface and crucial to maintaining cellular excitability in physiological conditions. Floriana* et al*. discovered that PRRT2 directly interacts with Na_v_1.2/Na_v_1.6 channels and acts as a negative regulator [11]. Indeed, excitatory synaptic inputs and inhibitory inputs are integrated at the axonal initial segment (AIS), where voltage-gated Na^+^ channels localize and their accumulation influences the threshold of action potential (AP). Importantly, Na_v_1.2/Na_v_1.6 channels are responsible for the generation of APs in excitatory neurons. Whereas, Na_v_1.1 channels which are responsible for the generation of APs in inhibitory neurons are not modulated by PRRT2. *In vitro* study, PRRT2 significantly decreased Na_v_1.2/Na_v_1.6 expression levels at the cell surface, without changes in the total level. When PRRT2 was co-expressed with Na_v_1.2 or Na_v_1.6, the Na^+^ current density was reduced and a slow-down in the recovery from inactivation was induced. Notably, iPSC-derived human neurons carrying homozygous c.649dupC showed a significant increase in Na^+^ current density and a decreased threshold for AP generation [11]. Mouse *Prrt2*-knockout (KO) cortical excitatory neurons also displayed a significantly increased length of the AIS, consistent with the increased intrinsic excitability. Significantly, the Na_v_1.2/Na_v_1.6 channels are encoded by epilepsy-associated ion channel genes *SCN2A* and *SCN8A* [36]. Mutations in *SCN9A*, another epilepsy-associated gene encoding Na_v_1.7, also contribute to an increase in seizures and show distinct sensitivity to OXC [37]. CBZ and OXC, targeting voltage-gated Na^+^ channels, have been verified to be highly effective in PKD, but incomplete remission was still reported in a few *PRRT2*-linked PKD patients [29]. It suggests that distinct sensitivity to CBZ and OXC may be associated with different types of mutations as well. Hence, the functional interactions between PRRT2 and Na_v_1.2/Na_v_1.6 channels and the clinical characteristics of PKD highly prove that PKD is an ionic channelopathy.

In addition to these Na^+^ channels, a pulldown-based proteomic approach identified the α1 and the α3 subunits of Na^+^/K^+^ ATPase pump (NKA) as major PRRT2-binding proteins in mouse brains. NKA mainly contributes to the resting potential and is essential for the ion gradient maintenance across the plasma membrane. Intriguingly, the lack of PRRT2 impaired the pump activity under conditions of neuronal stimulation, but not the resting conditions. Besides, NKA could provide intrinsic negative feedback to inhibit neural excitability after prolonged firing activity, as the result of increased intracellular Na^+^. In primary hippocampal neurons, PRRT2 deletion caused a significant decrease in pump activity and reduced the magnitude of NKA-dependent afterhyperpolarization which was elicited with a train of APs [35]. The α1 and the α3 subunits of NKA are encoded by *ATP1A1* and *ATP1A3* which are linked to epilepsy disorders [38, 39]. It hints that the paroxysmal manifestation may be associated with abnormal ion transporter functions. Overall, these studies indicate that PRRT2 exerts an inhibitory control of intrinsic excitability in neurons. The lack of PRRT2 leads to disturbed cellular excitability and network instability.

In recent years, various studies indicate the occurrence of PKD might be associated with abnormal synaptic function. PNKD, which has similar symptoms to PKD, is also a paroxysmal movement disorder precipitated by coffee, alcohol, and stress. The protein encoded by the causative gene *MR-1* localizes at synapses and regulates neurotransmitter release as well [40]. Then, these two paroxysmal movement disorders, PKD and PNKD were suspected as synaptopathies [10]. One study reported that the level of excitatory amino acids was higher in the plasma samples of PKD patients, consistent with increased concentration of glutamate (excitatory amino acid) in the culture medium of neurons following *Prrt2* knockdown [41]. Generally, PRRT2 is localized at axonal domains and presynaptic structures in neurons [18, 42, 43], and modulates the process of synaptic transmission (Fig. 3A). Between neurons, chemical communication is common in the mammalian central nervous system (CNS) and occurs at synapse. Presynaptic release of neurotransmitters is mediated by a three-step process: (1) synaptic vesicle (SV) tethering, docking at the active zone; (2) SV priming and forming the nucleus of synaptic soluble N-ethylmaleimide sensitive factor attachment protein receptor (SNARE) complex including three molecules, syntaxin1a (STX1A), SNAP-25 and synaptobrevin-2 (VAMP2); (3) Ca^2+^-triggered fusion [44]. A significantly higher number of docked synaptic vesicles in release sites in sections from *Prrt2*-KO mice were discovered [18, 43]. In the presynaptic structure, PRRT2 directly interacts with presynaptic proteins including the SNARE complex [18, 41, 45, 46], Ca^2+^ sensors synaptotagmins1/2 (Syt1/2) [42], and P/Q-type presynaptic voltage-gated Ca^2+^ channels (VGCCs) [47] which involves in the normal synaptic transmission.

Several studies found that PRRT2 negatively modulated SNARE complex assembly [18, 46, 48]. Assembled SNARE complex was significantly increased in the absence of PRRT2, accompanied by a reduction in the amount of STX1A, SNAP-25, and VAMP2. Coleman *et al.* showed that PRRT2 negatively modulated the SNARE-mediated vesicle fusion process in *vitro* reconstituted single vesicle and bulk fusion assays and PRRT2 acted primarily to regulate the priming process [46]. Meanwhile, the process of neurotransmitter release was also investigated to analyze the roles of PRRT2. In primary neurons, defective PRRT2 impaired synchronous release attributing to either the insensitivity of Ca^2+^ sensors Syt1/2 or a decreased VGCCs mediating Ca^2+^ influx [42, 47], and then increased facilitation of excitatory transmission. Nevertheless, the asynchronous release was relatively preserved, and SV priming was not affected [42]. Disparate study models and methods may account for this significant divergence in synaptic transmission. Interaction between PRRT2 with Intersectin1, an intracellular protein involved in synaptic vesicle cycling, was also shown in one study, while the detailed mechanisms of how PRRT2 influences their function remain to be elucidated [49]. Moreover, the subcellular location of PRRT2 has been controversial. Most studies indicated that PRRT2 existed only on the pre-synaptic membranes, but PRRT2 also presented on the post-membrane in a few studies. The level of an important subunit of the AMPA receptor GRIA1 was significantly higher upon co-expression with mutant PRRT2 [41, 50]. Although the role of PRRT2 in regulating neurotransmission is still under debate due to the conflicting results of recent studies, its loss of function leading to hyperexcitability is largely convincing. Myoclonus dystonia syndrome (MDS), which has a similar phenotype to dystonia in PKD, is caused by mutations in the *SGCE* gene. Loss of function of SGCE can lead to excessive excitatory synapses that may ultimately contribute to MDS [51]. Taken together, these results indicate that synaptic transmission between neurons may be changed in PKD, but definite proof still needs to be provided.

It seems that disturbed signal transmission in neurons is highly related to PKD. Loss-of-function of PRRT in neurons leading to hyperexcitability provides a simple and straightforward explanation for the paroxysmal manifestations observed in PKD patients. But PKD is a channelopathy or a synaptopathy or both? Interestingly, according to basic research, PRRT2 interacts with a variety of proteins in neurons and then regulates signal transmission from different standpoints. The various clinical phenotypes caused by *PRRT2* mutation may be associated with its rich functions. Which one is the dominant dysfunction *in vivo* contributing to PKD? Via interaction of the N-terminal proline-rich region with the synaptic SNARE proteins, PRRT2 regulates SNARE-mediated synaptic transmission [46]. PKD-associated *PRRT2* mutations disrupt the SNARE-moderating function and then result in PKD. This conclusion is disputable because missense mutations located at the N-terminal of *PRRT2* are mostly benign or likely benign according to the result of pathogenicity classification [17]. Therefore, the connection between the changed function of the SNARE complex and *PRRT2* linked-PKD should be further explained. Besides, electrical signals could be transformed into chemical signals at synapses. The high level of amino acids in PKD might be the secondary result of changed ionic channels and increased electrical signal conduction. In future research, experiments about the effect on ion channels and synaptic transmission in PKD models should be relatively independent and exclude interference with each other.

### Pathogenic Key Area in Cerebellum

The pathogenic area in PKD has been explored for many years. Researchers attempted to deepen the understanding of PKD by means of methods. Abnormalities of perfusion in the basal ganglia, thalamus, and cortex were observed in PKD patients through cerebral blood perfusion examination [52–54]. Moreover, in functional magnetic resonance imaging (fMRI) and diffusion tensor imaging (DTI) studies, microstructural alternations in the pre-supplementary motor area (preSMA) and the right opercular part of inferior frontal gyrus (IFGoperc) were found, and functional and structural connectivity between the thalamic nuclei and the cortex was significantly enhanced [55, 56]. In previous studies, 3T MRI was used to examine the structural and functional alteration in PKD. These days, a stronger field 7T MRI with higher resolution and signal-to-noise may be more sensitive to detect functional alterations and can be applied in the following research [57]. In general, according to the findings in neuroimaging studies, abnormalities in the basal ganglia-thalamo-cortical circuit in PKD have been suspected for a long time. However, little basic research into the deeper pathophysiologic mechanism of this basal ganglia-thalamocortical circuit was conducted. The lack of reliable animal models impeded the progress of understanding the mechanisms underlying PKD. Initially, Michetti *et al*. characterized the paroxysmal phenotype of *Prrt2* KO mice which was generated by a “knockout first” targeting strategy. Whereas, no direct evidence showed that any abnormal brain area was involved in the occurrence of dyskinesia attacks [58]. Instead, in 2018, Tan and colleagues generated mouse models with global and cerebellum-specific Prrt2 truncation, but not forebrain, displaying dyskinesia attacks resembling human PKD. Unlike previous methods, *Prrt2*^Stop^ knock-in mutant mice were generated by CRISPR/Cas-mediated genome editing, and *Prrt2* conditional KO mice were generated by Cre/*loxp* technology. The behavioral phenotypes in mutant mice were triggered by generalized seizures, hyperthermia, or optogenetic stimulation [18]. Generating appropriate experimental mouse models mimicking PKD has greatly promoted the studies of pathogenesis. In addition to these mouse models, rat models have been applied in research about PKD occasionally [48]. Whereas, neuronal circuits regulating motor function between humans and rodents vary largely. Thus, non-human primates and brain organoids can be considered as new models to explore these issues in further research. In short, these days, the cerebellum is widely recognized as the key area in the pathogenesis of PKD.

The cerebellum is an important center in the regulation of movement, where afferent fibers and efferent fibers are enriched. The highest expression level of Prrt2 in the brain occurs in the molecular layer of the cerebellar cortex. *In-situ* hybridization confirmed that Prrt2 was only expressed in granule cells (GCs) which account for most neurons in the cerebellar cortex and localized at the pre-synaptic space, but not in Purkinje cells (PCs) [43]. Notably, conditional knockout of Prrt2 in GCs was sufficient to induce dyskinesia attacks under hyperthermia stimulation [18]. It suggests that GCs in the cerebellum are center sites in the pathogenesis of PKD. GCs represent the input stage of the cerebellum. Via mossy fibers (MFs) originating from pre-cerebellar nuclei in the brainstem and spinal cord, GCs receive enriched signals including sensory and motor information. By electrophysiological recordings of GCs paired with MF electrical stimulation, synaptic transmission at MF-GCs synapses was not affected in *Prrt2* KO acute cerebellar slices [59]. All that matters is that Prrt2 deficiency affected the intrinsic excitability of GCs activity by increasing the expression of the Na^+^ channel and the length of the AIS [59]. Whereas, when lacking Prrt2, the effect in synaptic transmission at parallel fiber-Purkinje cell (excitatory synapses) in the cerebellar molecular layer was discrepant, increased, or decreased facilitation [18, 43]. Supposedly, different stimulation conditions cause these variable lab results.

A breakthrough study showed that Prrt2 deficiency facilitated the induction of spreading depolarization (SD) in the cerebellar cortex and dyskinesia under KCl stimulation. Cerebellar SD was highly associated with PKD since inhibition of SD prevented the occurrence of dyskinetic movements. In *Prrt2*-deficient mice, blocking the Na_v_ channels reduced excessive parallel fiber excitability and prevented the cerebellar SD and the following dyskinesia. Prrt2 deficiency may facilitate the generation of SD by reducing prolonged inactivation of Na_v_1.2 and Na_v_1.6 channels, leading to increased excitability among cerebellar GCs. Then, cerebellar SD caused a firing block of Purkinje cells (PCs) [60]. Significantly, the duration of attacks (often tens of seconds) in *Prrt2*-deficient mice overlapped with cerebellar SD-induced firing interruption of the PCs. PCs are the sole output neurons of the cerebellar cortex and send inhibitory projections to the deep cerebellar nuclei (DCN). Due to the pathologic GCs and PCs input, the cerebellar SD may lead to abnormal inhibition of the DCN. The firing rates of the DCN neurons were increased before they dramatically decreased, and the resultant aberrant firing patterns in DCN were temporally and tightly coupled with the dyskinetic episodes. These clues indicate a high correlation between the disruption of PC and DCN neurons and the occurrence of dyskinetic movements [60]. This pathway (GC-PC-DCN) deficit in the cerebellum (Fig. 3B) and pathological output from the cerebellum contribute to the motor phenotype seen in PKD.

The cerebellum is elucidated as a necessary locus for the generation of dyskinetic movements, whereas the role of other brain areas, such as the cortex, still cannot be neglected in PKD. In basic research, studies on mice revealed that Prrt2 was expressed throughout the CNS, with especially high levels in the cerebral cortex, hippocampus, basal ganglia, and cerebellum [4]. Mo *et al.* showed that Prrt2 deficiency leads to disrupted synaptic function in the primary motor cortex-induced PKD, although this conclusion has not been adequately approved. In the *Prrt2* KO rats, the level of SNARE elements and Syt1 rose significantly, and assembly of SNARE complex was also increased in the M1 cortex, but not in the cerebellar or hippocampal tissues [48]. Differently, Prrt2 deficiency facilitated the generation of SD and increased excitability among cerebellar GCs in the cerebellum by influencing Na_v_1.2 and Na_v_1.6 channels rather than Ca^2+^-dependent neurotransmitter release [60]. This result hints that the dysfunction of Na^+^ channels in the cerebellum is closely interlinked with PKD. Perhaps the dominant function of PRRT2 varies in different brain areas. Moreover, it is possible that dysfunction in various brain areas induces PKD together.

In terms of anatomy, the axons of DCN project to primary thalamic nuclei. Whether DCN-thalamic projections are problematic in PKD is of concern. Remarkably, through neuroimaging methods, other than structural abnormalities of the cerebellum, two newest studies indicated that dysconnectivity of the cerebello-thalamic circuit also contributed to the pathogenesis [61, 62]. Significantly, a primary PKD was cured after he accepted staged bilateral thalamotomy [63]. We suppose that abnormal neural network excitability in the cerebral cortex and cerebellum relay towards the thalamus and secondary alteration occurs in the thalamus. Breaking off turbulence in the thalamus which comes from the upper pathogenic area is sufficient to terminate the abnormity. Therefore, the thalamus may not be a primary pathogenic region but an important output node. The projections from DCN to the thalamus nuclei are enriched. For instance, one study showed that stimulation at the cerebellar interposed nucleus (IpN), one of the DCN, induced monosynaptic evoked potentials in the ventrolateral thalamus nuclei but not the contralateral region [64]. So, the specific pathogenic pathway from the cerebellum to the thalamus in PKD is still unclear. Various neurotropic viruses, spreading among synaptically-linked neurons, have been widely used to map neural networks. These methods can be considered to define the neural circuit in the pathogenesis of PKD [65]. Another possible mechanism is that the thalamus is also a considerable pathogenic area in the pathogenesis of PKD. Whereas, the alternative functions in the thalamus should be further studied. Based on the evidence above, disturbance of the cerebello-thalamo-cortical circuit may be highly associated with the occurrence of PKD. Functional connectivity and structural connectivity in the brain are complex. Furthermore, the connection of abnormal cerebellum and abnormal basal ganglia-thalamo-cortical circuit in the pathogenesis of PKD has been built up. Further research is needed to confirm the relationship between these brain areas and PKD.

## Conclusion and Perspective

The pathogenesis of PKD is complex and our understanding of it remains incomplete. *PRRT2* gene was identified as the major causative gene in PKD. Ion channels, ion transporters, and synaptic transmission in neurons are all affected by defective PRRT2. Among them, the interaction with the Na^+^ channel explains well why PKD patients have excellent responses to CBZ and strongly confirms that PKD is an ionic channelopathy. Priming and neurotransmitter release processes in synaptic transmission are also influenced by PRRT2, but the experiment results have been inconsistent in different research so far. The guess of synaptopathy needs to be verified by dealing with conflicting lab results. Significantly, the cerebellum has been regarded as the primary locus for the generation of dyskinetic movements in recent years. Nevertheless, in the progression of PKD, abnormal basal ganglia-thalamocortical circuits and cerebellar-thalamic circuits were frequently discovered by neuroimaging methods. Considering that secondary alteration or compensated change rather than root cause could also be found in PKD patients through neuroimaging methods, these brain areas in the pathogenesis of PKD need to be further identified in appropriate biological PKD models.

Despite extensive efforts to identify the mechanisms related to PRRT2 dysfunction, the reason why symptoms become less severe with age in PKD remains unclear. More than that, it is peculiar that the number of male patients is higher in PKD. By performing whole-cell patch clamp recordings, one study showed that male and female medial preoptic area (mPOA) neurons differ electrophysiologically [66]. Maybe disturbed neurons in PKD also have different electrophysiological properties between male and female patients. In the following experiments, sex differences should be attached to importance. The causative gene *TMEM151A* found recently may prompt researchers to explore the physiological function of the TMEM151A protein. Notably, the level of Tmem151a was relatively low during the embryonic period, markedly increased during postnatal stages, peaked at postnatal day 14, and declined in adulthood, very similar to the expression pattern of Prrt2 [5]. We speculate that the abnormal function may vary with aging and disappear in adult PKD patients. Even if variants lead to loss of function, they will not cause PKD during adulthood. The effect of age is needed to be considered in the pathophysiologic mechanisms of PKD in future research. PKD patients with *TMEM151A* variants also responded well to CBZ, but occasional attacks or sensory aura remain [29]. It suggests that TMEM151A may also affect ion channels, while some additional functions are not the same as PRRT2. TMEM151A localizes at ER where stores intracellular Ca^2+^ and plays an important role in intracellular Ca^2+^ mobilization and dynamics [5]. It prompts us to identify the relationship between TMEM151A and synaptic function. In addition, real-time PCR and in situ hybridization analyses revealed that *Tmem151a* was widely expressed in the CNS and was highly expressed in the cerebral cortex, thalamus, hippocampus, spinal cord, and brainstem. Unlike *Prrt2*, *Tmem151a* was mainly expressed in white matter, not in the cerebellar cortex [5]. Thus, the key area mediating the function of TMEM151A in controlling movement may be different from that of PRRT2. Gaining insights into both PRRT2 and TMEM151A function is of primary importance to understand the pathogenesis of PKD and then improve treatment in the future.

## References

[1] Kertesz A (1967). Paroxysmal kinesigenic choreoathetosis: An entity within the paroxysmal choreoathetosis syndrome. Description of 10 cases, including autopsied. Neurology.

[2] Chinese Society of Neurogenetics (2022). Neurogenetics Group of Neurology Branch of Chinese Medical Doctor Association. Chinese guidelines for diagnosis and treatment of paroxysmal kinesigenic dyskinesia. Chin J Neurol.

[3] Spacey S, Adams P (1993). Familial paroxysmal kinesigenic dyskinesia - RETIRED CHAPTER, FOR HISTORICAL REFERENCE ONLY.

[4] Chen WJ, Lin Y, Xiong ZQ, Wei W, Ni W, Tan GH (2011). Exome sequencing identifies truncating mutations in PRRT2 that cause paroxysmal kinesigenic dyskinesia. Nat Genet.

[5] Li HF, Chen YL, Zhuang L, Chen DF, Ke HZ, Luo WJ (2021). TMEM151A variants cause paroxysmal kinesigenic dyskinesia. Cell Discov.

[6] Wang HX, Li HF, Liu GL, Wen XD, Wu ZY (2016). Mutation analysis of MR-1, SLC2A1, and CLCN1 in 28 PRRT2-negative paroxysmal kinesigenic dyskinesia patients. Chin Med J (Engl).

[7] Yin XM, Lin JH, Cao L, Zhang TM, Zeng S, Zhang KL (2018). Familial paroxysmal kinesigenic dyskinesia is associated with mutations in the *KCNA1* gene. Hum Mol Genet.

[8] Li HF, Chen WJ, Ni W, Wang KY, Liu GL, Wang N (2013). PRRT2 mutation correlated with phenotype of paroxysmal kinesigenic dyskinesia and drug response. Neurology.

[9] Li HF, Wu ZY (2013). PRRT2 mutations and PRRT2 disorders. Human Genet Embryol.

[10] Valtorta F, Benfenati F, Zara F, Meldolesi J (2016). PRRT2: From paroxysmal disorders to regulation of synaptic function. Trends Neurosci.

[11] Fruscione F, Valente P, Sterlini B, Romei A, Baldassari S, Fadda M (2018). PRRT2 controls neuronal excitability by negatively modulating Na+ channel 1.2/1.6 activity. Brain.

[12] Méneret A, Grabli D, Depienne C, Gaudebout C, Picard F, Dürr A (2012). PRRT2 mutations: A major cause of paroxysmal kinesigenic dyskinesia in the European population. Neurology.

[13] Huang XJ, Wang SG, Guo XN, Tian WT, Zhan FX, Zhu ZY (2020). The phenotypic and genetic spectrum of paroxysmal kinesigenic dyskinesia in China. Mov Disord.

[14] Li HF, Ni W, Xiong ZQ, Xu J, Wu ZY (2013). PRRT2 c.649dupC mutation derived from de novo in paroxysmal kinesigenic dyskinesia. CNS Neurosci Ther.

[15] Chen YL, Chen DF, Ke HZ, Zhao SY, Li HF, Wu ZY (2022). Paroxysmal kinesigenic dyskinesia caused by 16p11.2 microdeletion and related clinical features. Neurol Genet.

[16] Tsai MH, Nian FS, Hsu MH, Liu WS, Liu YT, Liu C (2019). PRRT2 missense mutations cluster near C-terminus and frequently lead to protein mislocalization. Epilepsia.

[17] Zhao SY, Li LX, Chen YL, Chen YJ, Liu GL, Dong HL (2020). Functional study and pathogenicity classification of PRRT2 missense variants in PRRT2-related disorders. CNS Neurosci Ther.

[18] Tan GH, Liu YY, Wang L, Li K, Zhang ZQ, Li HF (2018). PRRT2 deficiency induces paroxysmal kinesigenic dyskinesia by regulating synaptic transmission in cerebellum. Cell Res.

[19] Huang XJ, Wang T, Wang JL, Liu XL, Che XQ, Li J (2015). Paroxysmal kinesigenic dyskinesia: Clinical and genetic analyses of 110 patients. Neurology.

[20] Heron SE, Grinton BE, Kivity S, Afawi Z, Zuberi SM, Hughes JN (2012). PRRT2 mutations cause benign familial infantile epilepsy and infantile convulsions with choreoathetosis syndrome. Am J Hum Genet.

[21] Lee HY, Huang Y, Bruneau N, Roll P, Roberson EDO, Hermann M (2012). Mutations in the gene *PRRT2* cause paroxysmal kinesigenic dyskinesia with infantile convulsions. Cell Rep.

[22] Riant F, Roos C, Roubertie A, Barbance C, Hadjadj J, Auvin S (2022). Hemiplegic migraine associated with *PRRT2* variations: A clinical and genetic study. Neurology.

[23] Liu Q, Qi Z, Wan XH, Li JY, Shi L, Lu Q (2012). Mutations in PRRT2 result in paroxysmal dyskinesias with marked variability in clinical expression. J Med Genet.

[24] Dale RC, Gardiner A, Antony J, Houlden H (2012). Familial PRRT2 mutation with heterogeneous paroxysmal disorders including paroxysmal torticollis and hemiplegic migraine. Dev Med Child Neurol.

[25] Chen Y, Chen D, Zhao S, Liu G, Li H, Wu ZY (2021). Penetrance estimation of PRRT2 variants in paroxysmal kinesigenic dyskinesia and infantile convulsions. Front Med.

[26] Labate A, Tarantino P, Viri M, Mumoli L, Gagliardi M, Romeo A (2012). Homozygous c.649dupC mutation in PRRT2 worsens the BFIS/PKD phenotype with mental retardation, episodic ataxia, and absences. Epilepsia.

[27] Delcourt M, Riant F, Mancini J, Milh M, Navarro V, Roze E (2015). Severe phenotypic spectrum of biallelic mutations in*PRRT2*gene. J Neurol Neurosurg Psychiatry.

[28] De Gusmao CM, Silveira-Moriyama L (2019). Paroxysmal movement disorders - practical update on diagnosis and management. Expert Rev Neurother.

[29] Chen YL, Chen DF, Li HF, Wu ZY (2022). Features differ between paroxysmal kinesigenic dyskinesia patients with PRRT2 and TMEM151A variants. Mov Disord.

[30] Wirth T, Méneret A, Drouot N, Rudolf G, Lagha Boukbiza O, Chelly J (2022). *De novo* mutation in *TMEM151A* and paroxysmal kinesigenic dyskinesia. Mov Disord.

[31] Mounir Alaoui O, Charbonneau PF, Prin P, Mongin M, Choquer M, Damier P (2023). TMEM151A as an alternative to PRRT2 in paroxysmal kinesigenic dyskinesia: About three new cases. Parkinsonism Relat Disord.

[32] Huang HL, Zhang QX, Huang F, Long XY, Song Z, Xiao B (2023). *TMEM151A* variants associated with paroxysmal kinesigenic dyskinesia. Hum Genet.

[33] Tian WT, Zhan FX, Liu ZH, Liu Z, Liu Q, Guo XN (2022). TMEM151A variants cause paroxysmal kinesigenic dyskinesia: A large-sample study. Mov Disord.

[34] Li HF, Chen WJ, Ni W, Wu ZY (2014). Paroxysmal kinesigenic dyskinesia and myotonia congenita in the same family: Coexistence of a *PRRT2* mutation and two *CLCN1* mutations. Neurosci Bull.

[35] Sterlini B, Romei A, Parodi C, Aprile D, Oneto M, Aperia A (2021). An interaction between PRRT2 and Na^+^/K^+^ ATPase contributes to the control of neuronal excitability. Cell Death Dis.

[36] Wei F, Yan LM, Su T, He N, Lin ZJ, Wang J (2017). Ion channel genes and epilepsy: Functional alteration, pathogenic potential, and mechanism of epilepsy. Neurosci Bull.

[37] Zhang S, Zhang Z, Shen Y, Zhu Y, Du K, Guo J (2020). SCN9A epileptic encephalopathy mutations display a gain-of-function phenotype and distinct sensitivity to oxcarbazepine. Neurosci Bull.

[38] Schlingmann KP, Bandulik S, Mammen C, Tarailo-Graovac M, Holm R, Baumann M (2018). Germline *de novo* mutations in ATP1A1 cause renal hypomagnesemia, refractory seizures, and intellectual disability. Am J Hum Genet.

[39] Holm R, Toustrup-Jensen MS, Einholm AP, Schack VR, Andersen JP, Vilsen B (2016). Neurological disease mutations of α3 Na^+^, K^+^-ATPase: Structural and functional perspectives and rescue of compromised function. Biochim Biophys Acta.

[40] Shen Y, Ge WP, Li Y, Hirano A, Lee HY, Rohlmann A (2015). Protein mutated in paroxysmal dyskinesia interacts with the active zone protein RIM and suppresses synaptic vesicle exocytosis. Proc Natl Acad Sci USA.

[41] Li M, Niu F, Zhu X, Wu X, Shen N, Peng X (2015). PRRT2 mutant leads to dysfunction of glutamate signaling. Int J Mol Sci.

[42] Valente P, Castroflorio E, Rossi P, Fadda M, Sterlini B, Cervigni RI (2016). PRRT2 is a key component of the Ca(2+)-dependent neurotransmitter release machinery. Cell Rep.

[43] Calame DJ, Xiao J, Khan MM, Hollingsworth TJ, Xue Y, Person AL (2020). Presynaptic PRRT2 deficiency causes cerebellar dysfunction and paroxysmal kinesigenic dyskinesia. Neuroscience.

[44] Südhof TC (2014). The molecular machinery of neurotransmitter release (Nobel lecture). Angew Chem Int Ed Engl.

[45] Schwenk J, Harmel N, Brechet A, Zolles G, Berkefeld H, Müller CS (2012). High-resolution proteomics unravel architecture and molecular diversity of native AMPA receptor complexes. Neuron.

[46] Coleman J, Jouannot O, Ramakrishnan SK, Zanetti MN, Wang J, Salpietro V (2018). PRRT2 regulates synaptic fusion by directly modulating SNARE complex assembly. Cell Rep.

[47] Ferrante D, Sterlini B, Prestigio C, Marte A, Corradi A, Onofri F (2021). PRRT2 modulates presynaptic Ca^2+^ influx by interacting with P/Q-type channels. Cell Rep.

[48] Mo J, Wang B, Zhu X, Wu X, Liu Y (2019). PRRT2 deficiency induces paroxysmal kinesigenic dyskinesia by influencing synaptic function in the primary motor cortex of rats. Neurobiol Dis.

[49] Rossi P, Sterlini B, Castroflorio E, Marte A, Onofri F, Valtorta F (2016). A novel topology of proline-rich transmembrane protein 2 (prrt2): Hints for an intracellular function at the synapse. J Biol Chem.

[50] Liu YT, Nian FS, Chou WJ, Tai CY, Kwan SY, Chen C (2016). PRRT2 mutations lead to neuronal dysfunction and neurodevelopmental defects. Oncotarget.

[51] Li J, Liu Y, Li Q, Huang X, Zhou D, Xu H (2021). Mutation in ε-sarcoglycan induces a myoclonus-dystonia syndrome-like movement disorder in mice. Neurosci Bull.

[52] Ko CH, Kong CK, Ngai WT, Ma KM (2001). Ictal ^99m^Tc ECD SPECT in paroxysmal kinesigenic choreoathetosis. Pediatr Neurol.

[53] Shirane S, Sasaki M, Kogure D, Matsuda H, Hashimoto T (2001). Increased ictal perfusion of the thalamus in paroxysmal kinesigenic dyskinesia. J Neurol Neurosurg Psychiatry.

[54] Kim YD, Kim JS, Chung YA, Song IU, Oh YS, Chung SW (2011). Alteration of ictal and interictal perfusion in patients with paroxysmal kinesigenic dyskinesia. Neuropediatrics.

[55] Long Z, Xu Q, Miao HH, Yu Y, Ding MP, Chen H (2017). Thalamocortical dysconnectivity in paroxysmal kinesigenic dyskinesia: Combining functional magnetic resonance imaging and diffusion tensor imaging. Mov Disord.

[56] Li HF, Yang L, Yin D, Chen WJ, Liu GL, Ni W (2019). Associations between neuroanatomical abnormality and motor symptoms in paroxysmal kinesigenic dyskinesia. Parkinsonism Relat Disord.

[57] Xie JJ, Li XY, Dong Y, Chen C, Qu BY, Wang S (2023). Local and global abnormalities in pre-symptomatic Huntington’s disease revealed by 7T resting-state functional MRI. Neurosci Bull.

[58] Michetti C, Castroflorio E, Marchionni I, Forte N, Sterlini B, Binda F (2017). The PRRT2 knockout mouse recapitulates the neurological diseases associated with PRRT2 mutations. Neurobiol Dis.

[59] Binda F, Valente P, Marte A, Baldelli P, Benfenati F (2021). Increased responsiveness at the cerebellar input stage in the PRRT2 knockout model of paroxysmal kinesigenic dyskinesia. Neurobiol Dis.

[60] Lu B, Lou SS, Xu RS, Kong DL, Wu RJ, Zhang J (2021). Cerebellar spreading depolarization mediates paroxysmal movement disorder. Cell Rep.

[61] Ekmen A, Meneret A, Valabregue R, Beranger B, Worbe Y, Lamy JC (2022). Cerebellum dysfunction in patients with *PRRT2*-related paroxysmal dyskinesia. Neurology.

[62] Kim MK, Suh SI, Kim JH (2022). Cerebello-thalamofrontal dysconnectivity in paroxysmal kinesigenic dyskinesia: A resting-state fMRI study. Parkinsonism Relat Disord.

[63] Horisawa S, Sumi M, Akagawa H, Kawamata T, Taira T (2017). Thalamotomy for paroxysmal kinesigenic dyskinesias in a multiplex family. Eur J Neurol.

[64] Ma KY, Cai XY, Wang XT, Wang ZX, Huang WM, Wu ZY (2021). Three-dimensional heterogeneity of cerebellar interposed nucleus-recipient zones in the thalamic nuclei. Neurosci Bull.

[65] Li J, Liu T, Dong Y, Kondoh K, Lu Z (2019). Trans-synaptic neural circuit-tracing with neurotropic viruses. Neurosci Bull.

[66] Zhang W, Li SS, Han Y, Xu XH (2021). Sex differences in electrophysiological properties of mouse medial preoptic area neurons revealed by *in vitro* whole-cell recordings. Neurosci Bull.

